# Immune Checkpoint Inhibitors in Clear Cell Renal Cell Carcinoma (ccRCC)

**DOI:** 10.3390/ijms26125577

**Published:** 2025-06-11

**Authors:** Jacek Rysz, Janusz Ławiński, Beata Franczyk, Anna Gluba-Sagr

**Affiliations:** 1 Department of Nephrology, Hypertension and Family Medicine, Medical University of Lodz, 90-549 Lodz, Poland; jacek.rysz@umed.lodz.pl (J.R.); beata.franczyk-skora@umed.lodz.pl (B.F.); 2 Department of Medicine, Faculty of Medicine, University of Rzeszow, 35-959 Rzeszow, Poland

**Keywords:** renal cell carcinoma, immune checkpoint inhibitors, PD-1 Inhibitors, PD-L1 Inhibitor, clinical trials

## Abstract

Renal cell carcinoma (RCC) accounts for about 403,000 new cases and 175,000 deaths worldwide each year. Clear cell RCC (ccRCC), the most prevalent subtype, is often driven by genetic mutations, such as VHL inactivation, leading to angiogenesis and immune escape. Immune checkpoint inhibitors (ICIs) targeting PD-1, PD-L1, and CTLA-4 have transformed treatment paradigms, yet therapeutic resistance remains a critical challenge. The immunosuppressive nature of the tumor microenvironment (TME) in ccRCC plays a central role in limiting ICI efficacy. Emerging strategies aim to overcome resistance by targeting key components of the TME, including tumor-associated macrophages, regulatory T cells (Tregs), and cytokine signaling. Agents such as nivolumab, pembrolizumab, and ipilimumab have demonstrated the ability to restore T-cell activity and mitigate immune suppression, offering clinical benefit in metastatic ccRCC. However, response rates vary, highlighting the need for rational combination therapies. ICIs combined with VEGF inhibitors have shown promising outcomes in clinical trials, and novel regimens continue to be explored. Risk stratification and personalized treatment selection are increasingly important as the therapeutic landscape evolves. This review synthesizes current advances in immunotherapy for ccRCC, with a focus on mechanisms of resistance and innovative strategies to enhance immune responsiveness. A deeper understanding of TME modulation and strategic combination approaches is essential to improve survival and quality of life for patients with advanced ccRCC.

## 1. Introduction

Renal cell carcinoma (RCC) is a biologically and clinically heterogeneous disease that ranks among the most common malignancies worldwide, with clear cell RCC (ccRCC) accounting for approximately 70% of cases [1,2,3]. Despite advances in imaging and earlier diagnosis, up to 25–30% of patients still present with advanced or metastatic disease, which remains associated with poor long-term survival outcomes [4].

Historically, treatment for advanced RCC relied on cytokine-based therapies, including interferon-alpha (IFN-α) and interleukin-2 (IL-2), which produced limited efficacy and significant toxicity [5]. The mid-2000s marked a turning point with the introduction of targeted therapies, particularly vascular endothelial growth factor (VEGF) receptor tyrosine kinase inhibitors (TKIs) and mammalian target of rapamycin (mTOR) inhibitors. These agents extended progression-free survival but were ultimately limited by resistance mechanisms [6]. 

The inherently immunogenic nature of ccRCC, characterized by frequent inactivation of the von Hippel-Lindau (VHL) tumor suppressor gene, resulting in aberrant hypoxia-inducible factor 2α (HIF-2α) stabilization, and overproduction of pro-angiogenic factors such as VEGF and platelet-derived growth factor (PDGF), provided a strong biological rationale for immunotherapy [5,6,7]. Molecular alterations, including chromosomal 3p losses affecting genes such as *PBRM1* and *BAP1*, not only contribute to angiogenesis and tumor progression but also promote an immunosuppressive tumor microenvironment (TME) that facilitates immune evasion [3,8,9].

The development of checkpoint inhibitors (ICIs) was a major step forward in the management of advanced RCC. Agents targeting programmed cell death protein-1 (PD-1), programmed death-ligand 1 (PD-L1), and cytotoxic T-lymphocyte-associated protein 4 (CTLA-4) have redefined treatment paradigms [10,11]. Landmark trials, such as CheckMate 025 and CheckMate 214, demonstrated that ICIs, used alone or in combination with other immunotherapies or anti-angiogenic agents, significantly improved survival outcomes compared to traditional VEGF-targeted therapies [10,12]. However, not all patients benefit equally. Clinical responses to ICIs remain heterogeneous, with many patients experiencing either primary resistance or disease progression after an initial response. The intricate interplay between tumor genomics, angiogenesis, immune signaling, and the TME underscores the biological complexity of ccRCC and highlights the urgent need for predictive biomarkers and innovative combination therapies. 

This review aims to provide a comprehensive overview of the immunobiology of ccRCC, evaluate the clinical evidence supporting the immune checkpoint blockade, and examine emerging strategies to overcome resistance and enhance immunotherapy efficacy in advanced disease.

A literature search for this review was conducted using PubMed and Web of Science for articles published between January 2015 and March 2025. The search focused on studies related to ccRCC, ICIs, and TME. The keywords used included “renal cell carcinoma”, “clear cell RCC”, “immunotherapy”, “immune checkpoint inhibitors”, “PD-1”, “PD-L1”, “CTLA-4”, “combination therapy,” “resistance,” and “ICI clinical trials”. The inclusion criteria were as follows: peer-reviewed original research, clinical trials, and reviews relevant to immunotherapy and ccRCC, published in English. The exclusion criteria included non-peer-reviewed articles, case reports, and studies not directly related to the topic. Additional sources were identified by reviewing references from the key article.

## 2. Tumor Immunobiology of ccRCC

Understanding the role of T cells and TME is essential to deciphering the immune response in cancer, particularly in ccRCC. Under normal physiological conditions, immune responses are tightly regulated to achieve a balance between effective pathogen clearance and prevention of tissue damage [13]. This is orchestrated through both intrinsic mechanisms, such as inhibitory receptors on T cells, and extrinsic mechanisms, including the secretion of immunosuppressive cytokines by immune and non-immune cells. Clear cell renal cell carcinoma is characterized by notable immunogenicity and a complex TME that critically influences disease progression and therapeutic response [14]. Within this microenvironment, a wide array of immune and stromal cells interacts to modulate anti-tumor immunity. The immune system’s ability to recognize and eliminate malignant cells in ccRCC is frequently compromized by immunosuppressive mechanisms operating within the TME [15]. Cancer cells reinforce suppression mechanisms, resulting in a tumor microenvironment that impairs effective immune responses [16]. A hallmark of ccRCC is the infiltration of immune cells within the tumor. The TME in ccRCC is a heterogeneous milieu composed of tumor cells, stromal cells (such as fibroblasts and endothelial cells), extracellular matrix, and a variety of immune cells. Among these immune cells are tumor-infiltrating lymphocytes (TILs), macrophages, myeloid-derived suppressor cells (MDSCs), and various T cell subsets [15,17,18]. Anti-tumor immunity in this context is largely mediated by cytotoxic CD8+ T cells and M1-polarized macrophages, both of which facilitate tumor cell destruction. T cells, particularly CD8+ cytotoxic T cells, are central to mounting effective anti-tumor responses in ccRCC. Their activation is initiated by antigen-presenting cells (APCs) like dendritic cells, which process tumor antigens and present them via MHC molecules to naïve T cells [19]. Upon antigen recognition, T cells proliferate and differentiate into effector cells capable of inducing apoptosis in tumor cells through the release of perforin and granzymes. However, the immunosuppressive signals within the TME impair the function and survival of these effector T cells. Regulatory T cells (Tregs) actively suppress their activation and proliferation, while immunosuppressive cytokines such as transforming growth factor-beta (TGF-β) and interleukin-10 (IL-10) inhibit T cell activity, contributing to immune evasion [20]. Furthermore, the metabolic conditions of the TME, characterized by hypoxia, acidic pH, and nutrient deprivation, negatively affect T cell metabolism and function [21]. M2-like macrophages dominate the tumor-associated macrophage population and contribute to immune evasion by promoting angiogenesis and extracellular matrix remodeling. Additionally, structural elements of the TME, such as the extracellular matrix and abnormal vasculature, influence immune cell infiltration and localization, limiting immune surveillance. Additionally, the loss of VHL function results in the accumulation of hypoxia-inducible factors (HIFs), which drive the expression of pro-angiogenic and immunosuppressive mediators that shape the TME toward immune evasion. A key immune evasion mechanism in ccRCC is the upregulation of immune checkpoint molecules such as PD-1, PD-L1 (its ligand), and CTLA-4, which suppress T cell activity and contribute to T cell exhaustion [22]. While these pathways help maintain immune homeostasis under normal conditions, in cancer, they contribute to immune suppression by limiting T cell activation and function within the tumor site. Emerging research has also highlighted the role of other checkpoint molecules, such as lymphocyte-activation gene 3 (LAG-3), T-cell immunoglobulin and mucin domain 3 (TIM-3), and T lymphocyte attenuator (BTLA), in further dampening T cell responses in ccRCC [22]. The interplay between immune checkpoints and tumor-intrinsic factors such as VHL gene inactivation and angiogenesis underscores the complexity of the TME and its critical role in shaping immune responses [23]. The immunogenic nature of ccRCC, evidenced by the presence of tumor-associated antigens and substantial immune infiltration, has positioned it as a strong candidate for immune checkpoint inhibition therapy. The most extensively studied immune checkpoints in this context include the PD-1/PD-L1 axis and the CTLA-4 pathway. 

PD-1 is a type I transmembrane glycoprotein belonging to the CD28/CTLA-4 family, expressed on activated T cells, B cells, and natural killer (NK) cells [16,24]. The PD-1/PD-L1 pathway plays a crucial role in modulating immune responses by diminishing T cell activity in peripheral tissues during inflammation, thereby protecting healthy tissues from immune-related injury. However, within the tumor microenvironment, this regulatory mechanism contributes to T cell dysfunction and impaired immune responsiveness, ultimately weakening the ability of cytotoxic T cells to attack and eliminate malignant cells [16]. Structurally, PD-1 contains a single immunoglobulin-like variable (IgV) domain responsible for interacting with its ligands, PD-L1 and PD-L2, and two intracellular immunoreceptor tyrosine-based motifs. Upon ligand binding, these motifs recruit tyrosine phosphatases, such as Src homology 2 domain-containing Phosphatase-1 (SHP1) and SHP2, which inhibit downstream signaling of the T and B cell receptor pathways, thereby suppressing immune activation [16,25,26,27]. PD-L1 and PD-L2, the primary ligands for PD-1, are commonly found on both tumor cells and APCs within the tumor microenvironment. Under physiological conditions, these ligands, which are members of the B7 family of immune checkpoint molecules, are expressed at low levels on kidney epithelial cells and become upregulated in response to inflammatory stimuli [16,28,29]. Binding of PD-1 to PD-L1 initiates a cascade of inhibitory signals within the T cell, resulting in the downregulation of T cell receptor (TCR) activity. This leads to diminished T cell proliferation, reduced cytokine secretion, and ultimately, the induction of T cell exhaustion, a state where T cells lose their ability to effectively eliminate tumor cells [30,31]. This mechanism is a central contributor to the establishment of an immunosuppressive tumor microenvironment, undermining the immune system’s ability to mount a robust anti-tumor response. In RCC, tumors with PD-1-positive TILs are associated with more aggressive clinical features. Tumors harboring these cells are significantly associated with larger tumor size (*p* = 0.001), higher nuclear grade (*p* = 0.001), advanced tumor-node-metastasis (TNM) stage (*p* = 0.005), presence of coagulative necrosis (*p* = 0.027), and sarcomatoid differentiation (*p* = 0.008) compared to tumors lacking PD-1-positive TILs [32].

CTLA-4 is an inhibitory receptor primarily found on activated T cells and regulatory T cells [33]. It functions during the early stages of the immune response, particularly within lymphoid tissues, where it modulates the initiation of T cell activation. CTLA-4 competes with CD28 for binding to B7 family ligands (CD80 and CD86) on APCs, thereby limiting the co-stimulatory signals needed for full T cell activation. By delivering inhibitory signals that counterbalance CD28-mediated stimulation, CTLA-4 promotes immune tolerance and helps prevent autoimmunity [34]. Tumors can exploit this pathway by upregulating CTLA-4 expression or recruiting CTLA-4-expressing Tregs to suppress anti-tumor immunity. Inhibitors such as ipilimumab target CTLA-4, restoring effector T cell activation and proliferation with tumoricidal potential. However, while the CTLA-4 blockade has demonstrated long-lasting benefits in some cancer patients, it carries a heightened risk of immune-related adverse events (irAEs) due to systemic immune activation [35]. Both PD-1/PD-L1 and CTLA-4 are pivotal in modulating T cell responses within the tumor microenvironment.

Lymphocyte activation gene-3 (LAG-3, CD223) is another immune checkpoint protein expressed on activated Tregs and, to a lesser extent, on effector T cells (Teffs) [36,37]. Under physiological conditions, LAG-3 upregulation plays a role in preventing autoimmunity. However, in the tumor microenvironment, persistent antigen exposure leads to LAG-3 over-expression, which contributes to T cell exhaustion and impaired immune function [38].

TIM-3, an immunoglobulin and mucin domain-containing cell surface molecule, is another key negative regulator of immune responses [39]. It is expressed on tumor cells, Teffs, Tregs, endothelial cells, and dendritic cells [38]. Studies have shown that TIM-3 is often co-expressed with other immune checkpoint molecules, such as PD-1, LAG-3, and TIGIT, on both CD4+ and CD8+ T cells [40,41]. Sakuishi et al. [42] observed that TIM-3(+)PD-1(+) TILs represented a predominant and highly exhausted T cell population in murine solid tumors, exhibiting impaired proliferation and cytokine production (e.g., IL-2, tumor necrosis factor (TNF), IFN-γ). TIM-3 interacts with four distinct ligands: galectin-9, phosphatidylserine (PtdSer), high-mobility group protein B1 (HMGB1), and carcinoembryonic antigen-related cell adhesion molecule-1 (CEACAM-1), all of which are implicated in tumor progression [43]. The third ligand, HMGB1 (which can also be released by cancer cells), acts as a damage-associated molecular pattern (DAMP), further contributing to immune evasion [44]. High expression of TIM-3 was demonstrated to correlate with a poor prognosis of patients with solid cancers, including ccRCC [45]. 

Another immune checkpoint, indoleamine 2,3-dioxygenase 1 (IDO1), is the rate-limiting enzyme involved in the catabolism of tryptophan into kynurenine [46]. In the tumor microenvironment, IDO1 was suggested to contribute to immunosuppression by depleting tryptophan, an essential amino acid for T cell function, and by increasing kynurenine levels [47,48]. This metabolic shift can induce apoptosis and dysfunction in Teffs cells, while also promoting the generation of immune-tolerant dendritic cells and expansion of Tregs through activation of the aryl hydrocarbon receptor (AhR) [38]. Enhanced expression of this enzyme is associated with the differentiation of CD4+ T cells into immunosuppressive Tregs, inhibition of antitumoral T cell responses, and polarization of antigen-presenting cells toward a tolerogenic phenotype [49,50]. These immunosuppressive conditions contribute to the inability of the immune system to effectively detect and destroy tumor cells [51,52]. Numerous studies have linked higher IDO1 expression with poor overall survival in cancer patients, emphasizing its role as a negative prognostic marker and a potential therapeutic target [48,53]. 

## 3. Overview of Immune Checkpoint Inhibitors

Recent advances in immuno-oncology have focused on addressing resistance mechanisms by targeting additional immunosuppressive components within the TME. Current strategies under investigation include reprogramming tumor-associated macrophages toward an M1 phenotype, depleting Tregs, modulating cytokine signaling, and combining ICIs with agents that promote vascular normalization. These approaches aim to reshape the immune milieu, thereby enhancing the effectiveness of immunotherapy in ccRCC.

ICIs that block PD-1, PD-L1, and CTLA-4, such as nivolumab, pembrolizumab, and ipilimumab, have shown clinical benefit in metastatic ccRCC by reactivating T cells and partially reversing immune suppression [10,11]. Despite these advances, patients’ responses remain variable, with only a fraction experiencing durable remissions. This heterogeneity has underscored the need for combination therapies that not only target checkpoint pathways but also modulate the immunosuppressive TME through mechanisms such as Treg depletion, macrophage polarization, and vascular modulation.

The immune checkpoint blockade targeting PD-1, PD-L1, and CTLA-4 functions by disrupting inhibitory pathways that tumors exploit to evade anti-tumor detection and destruction [54]. PD-1 inhibitors, which are monoclonal antibodies, block the PD-1 receptor on T cells. Normally, when PD-1 binds with its ligands (PD-L1 or PD-L2) on tumor or immune cells, it delivers an inhibitory signal that suppresses T-cell activity, leading to T-cell exhaustion. Blocking this interaction restores T cell proliferation, cytokine secretion, and cytotoxic capacity, thereby enhancing tumor clearance [55]. In contrast, PD-L1 inhibitors act by preventing PD-L1 from binding to PD-1 on T cells. By blocking this interaction, PD-L1 inhibitors also prevent T-cell inhibition and exhaustion, leading to enhanced T-cell-mediated anti-tumor activity. Unlike PD-1 inhibitors, PD-L1 inhibitors leave PD-L2/PD-1 signaling intact, which may result in a different toxicity profile [56]. CTLA-4 inhibitors, on the other hand, enhance immune activation at an earlier stage. Normally, CTLA-4 competes with the co-stimulatory molecule CD28 for binding to B7 ligands (CD80 and CD86) on antigen-presenting cells [57]. Its blockade leads to increased T cell priming and expansion in lymphoid organs, thereby boosting the population of effector T cells capable of infiltrating tumors and executing anti-tumor functions [57].

Several ICIs, such as nivolumab and pembrolizumab, avelumab and atezolizumab, as well as ipilimumab, have been approved or are under investigation for the treatment of ccRCC. 

Nivolumab is a PD-1 inhibitor that blocks the interaction between PD-1 and its ligands, thereby restoring T cell function [16]. It has demonstrated significant clinical activity in patients with mRCC, particularly in those who have progressed on prior anti-angiogenic therapies. Based on results from the Phase 3 CheckMate-025 trial (NCT01668784), nivolumab was approved by the FDA as a second-line treatment for metastatic ccRCC [58]. 

Pembrolizumab, another PD-1-targeting monoclonal antibody, has shown promising results, particularly in combination regimens. It is commonly paired with axitinib or lenvatinib in first-line settings [59].

Atezolizumab is an anti-PD-L1 monoclonal antibody blocking its interaction with PD-1 and B7.1 receptors to restore T-cell function and promote anti-tumor immunity. Its clinical potential as monotherapy in mRCC has been explored in early-phase and randomized trials [33].

Avelumab, also targeting PD-L1, has been studied in combination with axitinib for advanced RCC and has demonstrated favorable clinical activity [59].

Ipilimumab, an anti-CTLA-4 antibody, enhances T cell priming by antagonizing the binding of CTLA-4 to CD80/CD86 and is often used in combination with nivolumab in patients with intermediate or poor-risk features [59].

**Figure 1:**
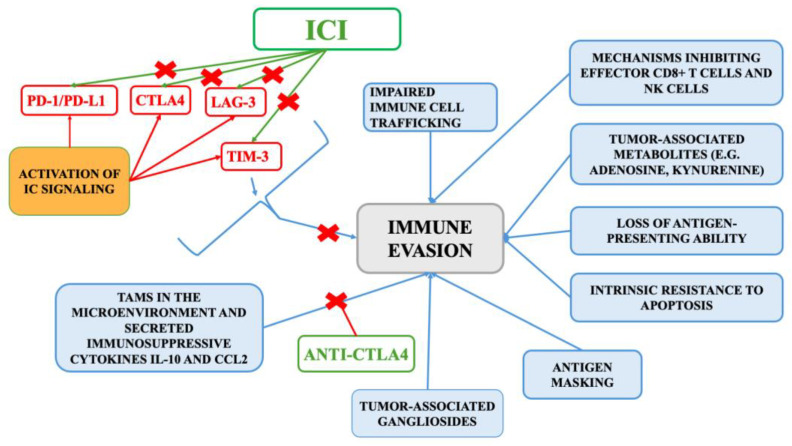
The mechanisms of immune evasion and the target of ICI.

In treatment selection, patient risk stratification plays a critical role. For individuals with favorable risk profiles, defined by the absence of adverse prognostic indicators, management may include active surveillance, metastasectomy, or systemic therapy [6]. Preferred systemic regimens include combinations such as axitinib with pembrolizumab, cabozantinib with nivolumab, and lenvatinib with pembrolizumab [60,61]. Monotherapy with VEGF receptor TKIs, such as pazopanib or sunitinib, may also be appropriate. For patients classified as intermediate or poor risk, based on the presence of one or more negative prognostic factors, first-line treatment commonly includes combinations (e.g., nivolumab with ipilimumab) or ICI-TKI regimens (e.g., axitinib plus pembrolizumab, cabozantinib plus nivolumab, or lenvatinib plus pembrolizumab). Axitinib combined with avelumab is another therapeutic alternative, while cabozantinib is frequently favored in this group for its broad anti-tumor activity [6,60,61]. The mechanisms of immune evasion and the target of ICI is presented on Figure 1.

Abbreviations: CCL2, C-C Motif Chemokine Ligand 2; CTLA-4, Cytotoxic T-Lymphocyte Antigen 4; IC, immune checkpoint; ICI, Immune Checkpoint Inhibitor; LAG-3, Lymphocyte-Activation Gene 3; NK, Natural Killer; PD-1, Programmed Cell Death Protein 1; PD-L1, Programmed Death-Ligand 1; TIM-3. T-cell Immunoglobulin and Mucin-Domain Containing-3

### 3.1. Monotherapy Clinical Trials

Clinical trials of ICI monotherapies in ccRCC, such as those involving atezolizumab, pembrolizumab, nivolumab, and avelumab, have demonstrated modest objective response rates (ORRs) ranging from 16% to 36% and progression-free survival (PFS) varying between 6 to 8 months in various phase I/II and phase II studies. These results highlight both the potential and limitations of monotherapy approaches in mccRCC.

#### 3.1.1. Nivolumab 

A small, prospective, phase II trial explored whether metastatic RCC patients, previously treated with anti-angiogenic agents, who respond to nivolumab, could safely pause treatment and resume only upon disease progression [62]. In this trial, patients who achieved at least a 10% decrease in tumor size after 12 weeks on nivolumab entered a treatment-free observation phase with therapy resumed only if the disease progressed. The treatment mode was assumed “feasible” if the acceptance rate was ≥80%. Out of 14 enrolled patients, only 1 required re-initiation of nivolumab, while the remaining participants maintained disease control off therapy for a median of 34 weeks (range, 16–53 weeks) [62]. These findings suggest that intermittent nivolumab dosing may preserve efficacy while potentially reducing cumulative toxicity and lowering healthcare costs. 

The open-label, phase III CheckMate 025 trial randomized patients with advanced RCC who had failed one or two prior lines of anti-angiogenic therapy to receive either nivolumab (3 mg/kg intravenously every 2 weeks) or everolimus (10 mg orally daily) [58]. Nivolumab significantly improved overall survival (OS) compared to everolimus, with a hazard ratio (HR) for death of 0.73 (98.5% CI, 0.57–0.93; *p* = 0.002), exceeding the pre-specified threshold for superiority (*p* ≤ 0.0148) [58]. The median OS was 25.0 months for nivolumab versus 19.6 months for everolimus. PFS did not differ significantly between the arms (4.6 months vs. 4.4 months; HR, 0.88; *p* = 0.11), but nivolumab achieved a higher objective response rate (23% vs. 4%). Additionally, nivolumab was associated with a lower incidence of grade 3 or 4 treatment-related adverse events (TRAEs) (19% vs. 37% for everolimus), underscoring its more favorable safety profile [58]. 

The final 5-year follow-up analysis of the CheckMate 025 confirmed the results [63]. At 60 months, 26% of patients treated with nivolumab remained alive compared to 18% in the everolimus arm, confirming a durable survival benefit and a favorable long-term safety, with no new safety signals reported. Overall, these findings establish nivolumab monotherapy as an effective and well-tolerated treatment option for patients with advanced or metastatic RCC following antiangiogenic therapy. The evidence supports its role not only in prolonging survival but also in offering an improved quality of life due to reduced toxicity [63].

Nivolumab received approval for monotherapy use in 2015 [64]. Since that milestone, combination immunotherapies have increasingly been integrated into clinical practice, marking the beginning of a new chapter in the treatment of renal cell carcinoma.

#### 3.1.2. Pembrolizumab 

Pembrolizumab, a monoclonal antibody targeting the PD-1 checkpoint, is under investigation both as an adjuvant therapy and as a first-line treatment in RCC, with particularly notable efficacy in clear-cell histology.

In the adjuvant setting, the randomized, double-blind, placebo-controlled phase III KEYNOTE-564 trial (NCT03142334) enrolled 994 patients with completely resected clear-cell RCC deemed at intermediate–high to high risk for recurrence, including those with resected metastases [65]. Participants were assigned to receive either pembrolizumab (200 mg IV every three weeks for up to 17 cycles) or placebo, starting within 12 weeks post-surgery and stratified by TNM stage and Fuhrman grade. At a median follow-up of 24.1 months (interim analysis), pembrolizumab reduced the risk of recurrence or death by 32% versus placebo, with 24-month disease-free survival (DFS) rates of 77.3% compared to 68.1% (HR for recurrence or death, 0.68; 95% CI, 0.53–0.87; *p* = 0.002). The estimated OS at 24 months also trended in favor of pembrolizumab (96.6% vs. 93.5%; HR for death, 0.54; 95% CI, 0.30–0.96). Grade ≥3 adverse events (AEs) occurred in 32.4% of the pembrolizumab arm versus 17.7% in the placebo arm, but no treatment-related deaths were reported. These findings highlight pembrolizumab’s role in prolonging DFS in patients at elevated risk of recurrence post-surgery [65].

In the metastatic setting, the phase II KEYNOTE-427 trial evaluated pembrolizumab monotherapy in 110 treatment-naïve patients with advanced RCC (cohort A) [66]. The study reported an ORR of 36.4%, including 3.6% complete responses (CR) and 32.7% partial responses (PR). Overall, 58.2% of patients (95% CI, 48.4–67.5) achieved disease control and 68.2% experienced tumor shrinkage, including 30.9% with ≥60% reduction in target lesions. The median PFS was 7.1 months (95% CI, 5.6–11.0), and the median duration of response extended to 18.9 months (range, 2.3–37.6+). Overall, 64.1% of responders maintained their response for at least 12 months. The median overall survival was not reached at the time of analysis, with 12-month and 24-month OS rates of 88.2% and 70.8%, respectively. Durable responses were observed across all International Metastatic RCC Database Consortium (IMDC) risk groups, including those with sarcomatoid differentiation and regardless of PD-L1 status.

The safety profile mirrored that seen in other malignancies treated with PD-1 inhibitors, with grade 3–5 TRAEs in 30% of patients, most commonly colitis and diarrhea, all managed according to established immunotherapy guidelines. IrAEs were managed according to standard guidelines, with corticosteroids employed in the majority of grade 2–4 cases.

Together, the findings from KEYNOTE-564 and KEYNOTE-427 demonstrate that pembrolizumab not only lowers the risk of recurrence when used as adjuvant therapy in high-risk RCC but also provides durable antitumor activity with manageable toxicity as monotherapy in the metastatic clear-cell setting.

#### 3.1.3. Avelumab

Avelumab, a fully human monoclonal antibody against PD-L1 IgG1, has demonstrated single-agent activity in metastatic RCC. In the phase Ib JAVELIN Solid tumor trial, treatment-naive patients (first-line, 1L) and those who had received one prior line of therapy (second-line, 2L) were given avelumab (10 mg/kg) intravenously every two weeks [67]. In the first-line cohort of 62 patients, the confirmed ORR rate was 16.1% with a median duration of response (DOR) of 9.9 months (95% CI, 2.8), not evaluable, and a median PFS of 8.3 months (95% CI, 5.5–9.5). Among 20 patients in the 2L subgroup, the ORR was 10.0%, the duration of response was not yet reached (95% CI, 6.9–not evaluable), and the median PFS was 5.6 months (95% CI, 2.3–9.6). The median OS had not been reached in the 1L group, whereas it was 16.9 months (95% CI, 8.3–not evaluable) in the 2L group.

TRAEs of any grade occurred in 82.3% of 1L patients and 70.0% of 2L patients, with grade ≥3 events in 12.9% and 5.0%, respectively, and no treatment-related deaths [67]. While these results confirm that avelumab monotherapy is active and well tolerated, the modest response rates have led to the development of combination strategies, most notably avelumab plus axitinib, which have since become first-line standards in advanced RCC. 

#### 3.1.4. Atezolizumab 

Atezolizumab monotherapy has also been evaluated in metastatic RCC. In a Phase Ia clinical trial including 63 patients with clear-cell and 7 with non-clear-cell metastatic RCC, treatment with atezolizumab was associated with manageable toxicity and signs of antitumor activity [68]. Responders exhibited decreases in circulating inflammatory and acute-phase proteins as well as higher baseline ratios of effector T-cell-to-regulatory T-cell gene expression, suggesting that pre-existing immune activation may predict benefit.

The randomized phase II IMmotion150 trial then compared the efficacy of atezolizumab alone, atezolizumab plus bevacizumab, and sunitinib, a standard VEGF-targeted therapy, in treatment-naïve patients [69]. In the intention-to-treat population (ITT), the median PFS was 6.1 months with atezolizumab monotherapy, 8.4 months with sunitinib, and 11.7 months with the combination of atezolizumab and bevacizumab. Among PD-L1-positive tumors, the median PFS was 5.5, 7.8, and 14.7 months, respectively.

ORRs followed a similar pattern. Atezolizumab monotherapy yielded an ORR of 25%, including 11% complete responses and 14% partial responses, 29% with sunitinib, and 32% with the combination therapy. In PD-L1-positive patients, the ORR with atezolizumab monotherapy was 28% versus 46% for the combination.

Importantly, tumors characterized by high pre-existing immune activity and low expression of myeloid-associated inflammatory genes (T_eff_^High^/Myeloid^Low^) derived the greatest benefit from atezolizumab alone, whereas those with a pronounced myeloid inflammatory signature (T_eff_^High^/Myeloid^High^) were less responsive, highlighting the critical role of the tumor microenvironment in shaping immunotherapy outcomes.

### 3.2. Combination Immunotherapy Trials

Given the complexity of immune regulation in ccRCC, combinatorial strategies are being investigated to improve therapeutic outcomes. These approaches include pairing ICIs with agents that target other components of the TME, such as therapies that deplete Tregs, reprogram tumor-associated macrophages toward an M1 phenotype, or normalize abnormal tumor vasculature. The goal is to simultaneously restore effective T cell-mediated immunity and dismantle the immunosuppressive barriers imposed by the TME.

#### 3.2.1. CHECKMATE 214: Nivolumab Plus and Ipilimumab Versus Sunitinib in Patients with Previously Untreated Advanced or Metastatic RCC

The CheckMate 214 was a landmark phase III, multicenter, open-label study that compared the dual immune checkpoint blockade with nivolumab (anti-PD-1) and ipilimumab (anti-CTLA-4) versus sunitinib in patients with previously untreated advanced or metastatic RCC [10]. A total of 1096 patients were randomized 1:1 to receive either combination immunotherapy (nivolumab: 3 mg/kg + ipilimumab: 1 mg/kg) every three weeks for four doses, followed by maintenance nivolumab every two weeks or sunitinib (50 mg daily, 4 weeks on, 2 weeks off). Among these, 847 patients had intermediate or poor prognostic features according to the IMDC criteria, forming the primary analysis population. The trial’s co-primary endpoints, OS, PFS, and ORR, focused on this subgroup. At a median follow-up of 25.2 months, combination immunotherapy demonstrated a significant OS benefit. The 18-month OS rate was 75% with nivolumab plus ipilimumab compared to 60% with sunitinib. The median OS was not reached in the immunotherapy arm versus 26.0 months in the sunitinib group (HR, 0.63; *p* < 0.001). The ORR was also significantly higher with dual immunotherapy (42% vs. 27%; *p* < 0.001), with CR observed in 9% versus 1% of patients, respectively. Although the median PFS was longer in the immunotherapy group (11.6 months vs. 8.4 months; HR, 0.82), it did not meet the predefined threshold for statistical significance [10]. In terms of safety, TRAEs occurred in 93% of patients receiving the combination therapy and 97% receiving sunitinib. Grade 3 or 4 TRAEs were less frequent in the immunotherapy group (46% vs. 63%). Discontinuation due to AEs was more common with combination immunotherapy (22% vs. 12%). Common high-grade AEs in the immunotherapy group included elevated lipase, fatigue, and diarrhea, while sunitinib was associated with hypertension, fatigue, and hand-foot syndrome. Most irAEs were manageable with corticosteroids and did not compromise long-term outcomes [10].

Extended follow-up has confirmed the durability of clinical benefits [70]. After a minimum of 4 years, OS remained significantly higher for immunotherapy in both the ITT population (HR 0.69) and in patients with intermediate/poor risk (I/P) (HR 0.65). Four-year PFS rates also favored nivolumab and ipilimumab (31.0% vs 17.3% in ITT and 32.7% vs 12.3% in I/P). ORRs remained higher with immunotherapy in both groups (ITT: 39.1% vs 32.4%; I/P: 41.9% vs 26.8%). Although sunitinib showed better OS and PFS outcomes in favorable-risk (FAV) patients, CR rates were still more frequent with the immunotherapy combination, including FAV patients (12.0% vs 6.5%). Furthermore, durable responses lasting four years or longer occurred more often in patients treated with nivolumab and ipilimumab, regardless of risk category. The 8-year follow-up (median 99.1 months) further supported the long-term efficacy of the dual checkpoint blockade. OS remained superior with immunotherapy in both ITT (HR 0.72) and I/P (HR 0.69) groups, though the survival difference was not significant in FAV patients (HR 0.82) [71]. Long-term PFS was better maintained in the ITT and I/P groups receiving nivolumab and ipilimumab, while sunitinib remained slightly superior in the FAV group. The ORR in the I/P subgroup continued to favor immunotherapy (42.4% vs 27.5%), though sunitinib showed higher ORR in FAV patients (51.6% vs 29.6%). Importantly, CR rates remained more frequent with combination immunotherapy across all subgroups. The median duration also favored the immunotherapy arm: 76.2 months vs. 25.1 months (ITT) and 82.8 months vs. 19.8 months (I/P). Safety remained consistent over time, with no new safety signals and manageable immune-related events [71].

The efficacy of this regimen is rooted in the complementary mechanism of its components: nivolumab restores T-cell activity by inhibiting PD-1, while ipilimumab enhances T-cell priming and proliferation by blocking CTLA-4. Together, these agents generate a sustained and effective anti-tumor immune response.

#### 3.2.2. KEYNOTE-426: Pembrolizumab Plus Axitinib vs. Sunitinib for First-Line Treatment of Advanced Renal Cell Carcinoma

The KEYNOTE-426 trial was a phase III, open-label, randomized trial evaluating the efficacy and safety of pembrolizumab combined with axitinib versus sunitinib as first-line therapy in patients with previously untreated advanced ccRCC. Conducted across 129 centers in 16 countries, the trial enrolled 861 patients who were randomly assigned in a 1:1 ratio to receive either pembrolizumab (200 mg IV every 3 weeks for up to 35 cycles) plus axitinib (5 mg orally twice daily) (432 patients) or sunitinib (50 mg orally once daily for 4 weeks on, 2 weeks off) (429 patients) [11].

The co-primary endpoints were OS and PFS, with the ORR as a key secondary endpoint. At the initial analysis, after a median follow-up of 12.8 months, the combination therapy demonstrated a significant improvement in outcomes compared to sunitinib [11]. The 12-month OS rate was 89.9% for the pembrolizumab–axitinib group versus 78.3% for the sunitinib group (HR for death, 0.53; 95% CI, 0.38–0.74; *p* < 0.0001). The median PFS was also longer with pembrolizumab–axitinib (15.1 vs. 11.1 months; HR, 0.69; 95% CI, 0.57–0.84; *p* < 0.001). The ORR was significantly higher in the pembrolizumab–axitinib group (59.3% vs. 35.7%; *p* < 0.001), with benefits observed across all IMDC risk categories and irrespective of PD-L1 status [11].

Extended follow-up analyses confirmed the durability of these benefits. At a median follow-up of 30.6 months, the median OS was not reached in the pembrolizumab–axitinib group versus 35.7 months in the sunitinib group (HR, 0.68; 95% CI, 0.55–0.85; *p* = 0.0003) [72]. The median PFS remained superior at 15.4 months vs. 11.1 months, respectively (HR, 0.71; 95% CI, 0.60–0.84; *p* < 0.0001) [72]. 

At the final protocol-specified analysis of the phase 3 KEYNOTE-426 trial, with a median follow-up of 43 months, the combination of pembrolizumab plus axitinib continued to demonstrate superior efficacy over sunitinib [73]. Specifically, the median OS (HR, 0.73; 95% CI, 0.60–0.88), PFS (HR, 0.68; 95% CI, 0.58–0.80), and ORR (60% vs. 40%) remained favorable. Additionally, the median DOR was extended in the combination arm, reaching 23.6 months versus 15.3 months in the sunitinib arm [73]. 

Grade 3 or higher treatment-emergent adverse events (TEAEs) occurred in 75.8% of patients receiving pembrolizumab–axitinib and 70.6% of those on sunitinib [11]. Common severe events included hypertension (22% vs. 20%), elevated alanine aminotransferase levels (13% vs. 3%), and diarrhea (11% vs. 5%). No new treatment-related deaths were reported beyond the interim analysis [72].

Health-related quality of life (HRQoL) was assessed using FKSI-DRS (Functional Assessment of Cancer Therapy–Kidney Symptom Index–Disease-Related Symptoms), EORTC QLQ-C30 (European Organisation for Research and Treatment of Cancer Quality of Life Questionnaire–Core 30), and EQ-5D VAS (EuroQol 5-Dimension Visual Analogue Scale) instruments [74]. Improvements from baseline in overall HRQoL were either better or not significantly different for pembrolizumab–axitinib compared to sunitinib across all instruments. Time to first deterioration and time to confirmed deterioration were mostly similar between arms [74]. 

#### 3.2.3. IMmotion151: A Phase III Trial of Atezolizumab Plus Bevacizumab Versus Sunitinib in Advanced Renal Cell Carcinoma

The IMmotion151 study was a multinational, open-label, Phase III trial that randomly assigned treatment-naïve patients with unresectable, locally advanced, or metastatic ccRCC to receive either atezolizumab (an anti-PD-L1 antibody) plus bevacizumab (an anti-VEGF antibody) or sunitinib. Eligible participants had no prior systemic therapy in either the metastatic or adjuvant setting and were enrolled at 152 academic and community sites across 21 countries from May 2015 to October 2016 [75]. Randomization was 1:1 to atezolizumab 1200 mg IV plus bevacizumab 15 mg/kg IV every 3 weeks (n = 454) or sunitinib 50 mg orally once daily on a 4-weeks-on/2-week-off schedule (n = 461). Treatment continued until radiographic progression, unacceptable toxicity, clinical decline, or withdrawal of consent. At baseline, 40% of tumors were PD-L1 positive [76]. After a median follow-up of 15 months for the primary PFS analysis, patients with PD-L1 positive disease achieved a median PFS of 11.2 months (95 % CI, 9.7–14.0) on the combination regimen versus 7.7 months (95 % CI, 6.9–9.2) with sunitinib (HR, 0.74; 95 % CI, 0.57–0.96; *p* = 0.0217). However, in the overall ITT population, the interim OS analysis showed no significant difference between arms (HR 0.93; 95% CI: 0.76–1.14) [76].

The incidence of grade 3–4 TRAEs was lower in the atezolizumab plus bevacizumab group (40%) compared to sunitinib (54%) and discontinuations due to toxicity were reported in 5% and 8% of patients, respectively [76].

With extended follow-up (median 24 months for OS), the median survival in the ITT cohort was 36.1 months for the combination arm versus 35.3 months with sunitinib, and in the PD-L1-positive subgroup, 38.7 versus 31.6 months, respectively [77]. However, exploratory transcriptomic analyses identified that tumors with T-effector/proliferative, proliferative, or small nucleolar RNA signatures derived a greater OS benefit (35.4 months vs. 21.2 months; HR, 0.70; 95 % CI, 0.50–0.98) [77].

Patient-reported outcomes (PROs), including symptom burden, interference with daily life, treatment-related bother, and overall quality of life, favored the atezolizumab plus bevacizumab arm with HRs ranging from 0.45 to 0.68 [78]. Assessment completion rates for PRO remained ≥70% through week 54, underscoring the regimen’s tolerability and impact on patient well-being.

#### 3.2.4. CheckMate 9ER: A Phase III Study of Nivolumab Plus Cabozantinib Versus Sunitinib in Treatment-Naïve Advanced Renal Cell Carcinoma

CheckMate 9ER was a worldwide, open-label, phase III trial that enrolled adults with untreated advanced or metastatic RCC to compare the efficacy and safety of the combination of nivolumab (a PD-1 checkpoint inhibitor) and cabozantinib (a VEGFR/MET/AXL tyrosine kinase inhibitor) against sunitinib monotherapy [79]. Patients were randomized 1:1 to receive 240 mg of nivolumab intravenously every two weeks with 40 mg of oral cabozantinib daily, or 50 mg of sunitinib orally once daily on a 4-weeks-on/2-week-off cycle. The trial’s primary endpoint was PFS, assessed by blinded independent central review. Secondary endpoints included the OS, ORR, and safety and exploratory HRQoL assessments.

Between 11 September 2017, and 14 May 2019, 651 patients were randomized (nivolumab + cabozantinib, n = 323; sunitinib n = 328) [79]. At a median follow-up of 18.1 months, the combination arm achieved a median PFS of 16.6 months (95% CI: 12.5–24.9), compared to 8.3 months (95% CI: 7.0–9.7) with sunitinib (HR 0.51; 95% CI: 0.41–0.64; *p* < 0.001). The estimated 12-month OS rate was 85.7% (95% CI: 81.3–89.1) for the nivolumab–cabozantinib cohort versus 75.6% (95% CI: 70.5–80.0) with sunitinib (HR for death, 0.60; 98.89% CI: 0.40–0.89; *p* = 0.001). The ORR was significantly higher with the combination (55.7% vs. 27.1%, *p* < 0.001), with a consistent benefit across pre-specified subgroups [79]. With data cutoff on 24 June 2021 (median follow-up 32.9 months; interquartile range [IQR] 30.4–35.9), the combination continued to demonstrate durable efficacy: the median OS was 37.7 months (95% CI: 35.5–not estimable) versus 34.3 months (95% CI: 29.0–not estimable) for sunitinib (HR 0.70; 95% CI: 0.55–0.90; *p* = 0.0043) and the updated median PFS remained 16.6 months (95% CI: 12.8–19.8) versus 8.3 months (95% CI: 7.0–9.7) (HR 0.56; 95% CI: 0.46–0.68; *p* < 0.0001) [80].

Safety analyses revealed similar overall rates of grade 3–4 AEs of any cause (75.3% combination vs. 70.6% sunitinib), though grade 3–4 TRAEs occurred more often with nivolumab–cabozantinib (65% vs. 54%) [79,80]. The most frequent severe AEs in the combination arm were hypertension (13%), palmar–plantar erythrodysesthesia (8%), and diarrhea (7%). Serious grade 3–4 TRAEs affected 22% of patients on the combination versus 10% with sunitinib, and 19.7% discontinued at least one trial drug due to toxicity (5.6% stopped both agents) [79,80]. One treatment-related death (sudden death) was reported in the sunitinib arm. Patient-reported outcomes favored the combination therapy, showing improvements in global HRQoL, symptom burden, and functional interference compared to sunitinib, with fewer treatment-related bothers and sustained completion rates (≥70% through week 54). An exposure–response analysis indicated that adding cabozantinib to nivolumab enhanced efficacy relative to nivolumab alone (PFS: HR 0.38; 95% CI: 0.31–0.47; OS: HR 0.63; 95% CI: 0.46–0.85) but also increased the risk of grade ≥2 immune-mediated adverse events (IMAEs) (HR 2.19; 95% CI: 1.79–2.67) [81]. Favorable outcomes correlated with lower nivolumab clearance, male sex, higher baseline body weight, and a Karnofsky performance status of 100, while geographic region and poor-risk IMDC scores were associated with worse OS. Cabozantinib exposure notably drove gastrointestinal and hepatic immune-mediated toxicities. Notably, cabozantinib contributed significantly to grade ≥2 IMAEs, especially gastrointestinal and hepatic events. Pharmacokinetic modeling supported equivalent benefit–risk profiles for nivolumab and predicted similar benefit–risk profiles for dosing schedules of 240 mg of nivolumab every 2 weeks versus 480 mg every 4 weeks, both combined with cabozantinib [81].

In a separate exploratory cohort of a discontinued arm of the CheckMate 9ER trial, 50 patients received a triplet induction of nivolumab (3 mg/kg) plus ipilimumab (1 mg/kg) every 3 weeks for four cycles alongside daily cabozantinib (40 mg), followed by maintenance nivolumab (240 mg every 2 weeks) and cabozantinib [82]. After a median follow-up of 39.1 months (range: 33.4–44.5), the median PFS was 9.9 months by blinded central review (95% CI: 5.7–16.8) and 13.9 months by investigator assessment (95% CI: 7.3–24.7). The median OS reached 37.0 months (95% CI: 31.8–not estimable). The objective response rate was 44.0% (8.0% CR) per central review and 48.0% per investigator assessment (all partial responses). Grade 3–4 TRAEs occurred in 84.0% patients, predominantly manifested as elevated liver enzymes and hepatotoxicity, with grade 3–4 hepatic IMAEs affecting 40% of patients, though no treatment-related deaths were reported [82].

#### 3.2.5. JAVELIN Renal 101: A Phase 3 Study of Avelumab Plus Axitinib Versus Sunitinib in First-Line Advanced Renal Cell Carcinoma

The JAVELIN Renal 101 trial was a large, randomized, open-label, phase III study (NCT02684006) that enrolled 886 patients with previously untreated, advanced ccRCC to compare the efficacy and safety of avelumab in combination with axitinib versus sunitinib monotherapy [83]. Patients were randomly assigned in a 1:1 ratio to receive either avelumab (10 mg/kg intravenously every two weeks) together with axitinib (5 mg orally twice daily) or sunitinib at 50 mg orally once daily on a four-week-on, two-week-off schedule. The co-primary endpoints of the study were PFS and OS in the subset of patients whose tumors expressed PD-L1. Key secondary endpoints included PFS and OS in the overall population, ORR, and safety.

In total, 442 patients were assigned to the avelumab–axitinib arm and 444 were treated with sunitinib. Of these, 560 (63.2%) had PD-L1-positive tumors: 270 in the combination arm and 290 in the sunitinib arm [83]. In the PD-L1-positive subgroup, the median PFS was significantly longer with avelumab plus axitinib (13.8 months; 95% CI, 11.1, could not be estimated) than with sunitinib (7.2 months; 95% CI, 5.7–9.7), corresponding to a hazard ratio for progression or death of 0.61 (95% CI, 0.47–0.79; *p* < 0.001). In the overall population, the median PFS was also longer with the combination regimen (13.8 months; 95% CI, 11.1–could not be estimated) versus the sunitinib arm (8.4 months; 95% CI, 6.9–11.1), with an HR of 0.69 (95% CI, 0.56–0.84; *p* < 0.0001) [83].

At the time of the initial analysis, OS data were still immature but trended in favor of the avelumab–axitinib combination. In PD-L1-positive patients, the HR for death was 0.83 (95% CI, 0.60–1.15; *p* = 0.13), and in the overall cohort, the HR was 0.80 (95% CI, 0.62–1.03; *p* = 0.0392) [84]. The ORR in PD-L1-positive patients was markedly higher with the combination therapy (55.2%) than with sunitinib (25.5%) [83]. Safety profiles were similar: nearly all patients experienced TEAEs (99.5% in the avelumab–axitinib group and 99.3% in the sunitinib group), and grade 3 or higher AEs occurred in 71.2% and 71.5% of patients, respectively [83].

Subsequent biomarker analyses showed that neither PD-L1 expression nor PD-L1 expression or tumor mutational burden significantly predicted PFS in either treatment group [85]. However, exploratory genomic and immunologic profiling identified several gene expression signatures related to immune modulation and angiogenesis, certain mutational patterns, and specific HLA types that appeared to correlate with differential outcomes to the combined checkpoint inhibitor and tyrosine kinase inhibitor strategy [85].

A post hoc evaluation of 108 patients whose tumors had sarcomatoid features (particularly the aggressive RCC subtype), revealed that those treated with avelumab plus axitinib experienced improved PFS (HR 0.57; 95% CI, 0.33–1.00) and a higher ORR (46.8% vs. 21.3%), including complete responses in 4.3% of cases versus none in the sunitinib group [86]. Gene expression analysis in this subgroup revealed enrichment in regulatory T-cell and cancer-associated fibroblast pathways, elevated expression of CD274 and CD8A, and a predominance of The Cancer Genome Atlas m3 classification, suggesting potential biological drivers of enhanced response in sarcomatoid RCC [86].

#### 3.2.6. CLEAR Trial: Phase 3 Study Comparing Lenvatinib-Based Combinations Versus Sunitinib as First-Line Therapy for Advanced Renal Cell Carcinoma

The CLEAR trial was a global, open-label, phase III study that randomly assigned treatment-naïve patients with advanced RCC in a 1:1:1 ratio to one of three arms: lenvatinib plus everolimus, lenvatinib plus pembrolizumab, or sunitinib monotherapy [87]. In the combination arms, patients received either lenvatinib (18 mg orally once daily) plus everolimus (5 mg orally once daily) or lenvatinib (20 mg orally once daily) plus pembrolizumab (200 mg intravenously every 3 weeks); the control arm received sunitinib 50 mg daily on a 4-week-on/2-week-off schedule. The primary efficacy measure was PFS per RECIST 1.1 as adjudicated by an independent review committee, while OS and safety were key secondary endpoints.

A total of 1069 patients were randomized evenly across the three arms (355 to lenvatinib+pembrolizumab, 357 to lenvatinib+everolimus, and 357 to sunitinib) [87]. The lenvatinib–pembrolizumab arm demonstrated a marked improvement in the median PFS versus sunitinib (23.9 months vs. 9.2 months; HR for progression or death of 0.39 (95% CI, 0.32–0.49; *p* < 0.001) and lenvatinib–everolimus also significantly prolonged PFS compared with sunitinib (14.7 months vs. 9.2 months; HR, 0.65; 95% CI, 0.53–0.80; *p* < 0.001). In terms of OS, the lenvatinib–pembrolizumab combination conferred a survival advantage over sunitinib (HR for death, 0.66; 95% CI, 0.49–0.88; *p* = 0.005). However, no OS benefit was observed for the lenvatinib–everolimus arm compared to sunitinib (HR, 1.15; 95% CI, 0.88–1.50; *p* = 0.30).

Grade 3 or higher AE occurred in 82.4% of patients receiving lenvatinib–pembrolizumab, 83.1% of those on lenvatinib–everolimus, and 71.8% of the sunitinib cohort. The most common severe TEAEs (occurring in ≥10% of patients in any group) included hypertension, diarrhea, and elevated lipase levels across the three arms.

In hHRQOL assessments (median follow-up 12.9 months; lenvatinib–pembrolizumab showed more favorable mean changes from baseline than sunitinib in FKSI-DRS (−1.75 vs. −2.19), EORTC QLQ-C30 global health status/quality of life (GHS/QOL) score (−5.93 vs. −6.73), and EQ-5D visual analogue scale (VAS) (−4.96 vs. −6.64) [88]. Time to first deterioration did not differ significantly for FKSI-DRS (median 9.14 vs. 12.14 weeks in the sunitinib group; HR 1.13; 95% CI 0.94–1.35; log-rank *p* = 0.20) or EORTC QLQ-C30 GHS/QOL (12.00 vs. 9.14 weeks; HR 0.88; 95% CI 0.74–1.05; log-rank *p* = 0.17) but favored lenvatinib–pembrolizumab for EQ-5D VAS (9.43 vs. 9.14 weeks; HR 0.83; 95% CI 0.70–0.99; log-rank *p* = 0.041). When considering definitive deterioration, defined as a clinically meaningful and sustained decline in HRQOL, patients on lenvatinib plus pembrolizumab had a significantly longer time to definitive decline (114.29–134.14 weeks) compared with sunitinib (74.86–117.43 weeks). Importantly, none of the HRQOL measures favored sunitinib over the lenvatinib–pembrolizumab combination. By contrast, lenvatinib–everolimus and sunitinib showed either comparable or slightly better HRQOL outcomes with sunitinib [88].

#### 3.2.7. COSMIC-313: Phase 3 Trial of Cabozantinib in Combination with Nivolumab and Ipilimumab for Advanced Renal Cell Carcinoma

The COSMIC-313 is a multicenter, randomized, double-blind, placebo-controlled phase III trial comparing the efficacy and safety of the addition of cabozantinib to the standard nivolumab-ipilimumab regimen against nivolumab-ipilimumab plus placebo in treatment-naïve patients with advanced or metastatic ccRCC meeting intermediate or poor-risk IMDC criteria [89]. A total of 855 patients were enrolled at 180 sites worldwide and randomized 1:1 to either the experimental (cabozantinib + nivolumab + ipilimumab) or control arm (placebo + nivolumab + ipilimumab).

In the cabozantinib arm, patients received oral cabozantinib (40 mg daily) together with intravenous nivolumab (3 mg/kg) and ipilimumab (1 mg/kg) every three weeks for four cycles; thereafter, nivolumab maintenance therapy (480 mg every four weeks) continued for up to two years. The control group followed the identical nivolumab-ipilimumab schedule, substituting the placebo for cabozantinib. The primary efficacy endpoint was PFS, assessed via a blinded independent review committee according to RECIST v1.1, in the first 550 randomized patients. OS is a key secondary endpoint evaluated in the full cohort of 855 patients (428 in the experimental arm and 427 in the control arm).

Among the initial 550 patients (276 in the cabozantinib arm, 274 in the control group), the 12-month PFS rate was 57% with the triplet combination and 49% in the control group. The addition of cabozantinib to nivolumab and ipilimumab produced a statistically significant PFS benefit (HR 0.73; 95% CI, 0.57–0.94; *p* = 0.01). ORRs were also higher in the experimental group (43% vs. 36%). However, grade 3 or 4 adverse events were more frequent when cabozantinib was included (79% vs. 56% in the control group). Final OS data are still maturing and are being monitored across the full study population.

#### 3.2.8. RENOTORCH Phase III Trial: Toripalimab Plus Axitinib vs. Sunitinib in First-Line Treatment of Advanced RCC

The RENOTORCH is a randomized, open-label, multicenter phase III trial evaluating the efficacy and safety of toripalimab in combination with axitinib compared to sunitinib monotherapy as a first-line treatment for patients with unresectable or metastatic intermediate-/poor-risk RCC according to IMDC criteria [90]. A total of 421 patients were enrolled and equally randomized. In the experimental arm, patients received toripalimab (240 mg IV every three weeks) alongside axitinib (5 mg orally twice daily). In the control arm, sunitinib was administered at 50 mg orally once daily, either on a 4-weeks-on/2-week-off (6-week) schedule or an alternating 2-weeks-on/1-week-off (3-week) cycle, based on individual tolerability. The primary endpoint was investigator-blinded PFS, with secondary endpoints including investigator-assessed the PFS, ORR, OS, and safety. After a median observation of 14.6 months, the toripalimab-axitinib combination reduced the risk of disease progression or death by 35% compared to sunitinib (HR 0.65, 95% CI 0.49–0.86; *p* = 0.0028). The median PFS was 18.0 months in the combination cohort versus 9.8 months in the sunitinib arm. The IRC-assessed ORR was 56.7% with toripalimab-axitinib arm, markedly higher than the 30.8% seen with sunitinib (*p* < 0.0001). A trend toward improved OS also favored the combination therapy (HR 0.61, 95% CI 0.40–0.92). Grade ≥3 treatment-related adverse events were reported in 61.5% of patients receiving toripalimab-axitinib and 58.6% of those on sunitinib, indicating that both regimens had a manageable safety profile [90].

#### 3.2.9. CONTACT-03: Atezolizumab Combined with Cabozantinib Versus Cabozantinib Alone in Renal Cell Carcinoma Following Progression on Immune Checkpoint Inhibitors

The CONTACT-03 study was a phase III, multicenter, randomized trial conducted across 135 sites in 15 countries spanning Asia, Europe, North America, and South America [91]. It enrolled patients aged 18 years and older with locally advanced or metastatic RCC whose disease had progressed after treatment with immune checkpoint inhibitors. Participants were randomized in a 1:1 ratio to receive either a combination of atezolizumab (1200 mg intravenously every three weeks) and cabozantinib (60 mg orally once daily) or cabozantinib monotherapy. The co-primary endpoints were PFS, as assessed by blinded independent central review, and OS. Efficacy analyses were performed in the ITT population, while safety was evaluated in all patients who received at least one dose of the study treatment.

Between 28 July 2020, and 27 December 2021, 522 patients were randomized: 263 to the combination group and 259 to the cabozantinib-only arm [91]. At the data cutoff on 3 January 2023, the median follow-up duration was 15.2 months (IQR 10.7–19.3 months). Progression or death had occurred in 171 (65%) patients receiving the triplet regimen and 166 (64%) in the monotherapy group. The median PFS was 10.6 months (95% CI 9.8–12.3) with atezolizumab plus cabozantinib versus 10.8 months (95% CI 10.0–12.5) for cabozantinib monotherapy (HR 1.03; 95% CI 0.83–1.28; *p* = 0.78). The proportion of deaths was identical: 34% in each group, 89 patients in the combination group and 87 in the monotherapy arm. The median OS reached 25.7 months (95% CI 21.5, not evaluable) in the combination group and was not evaluable in the cabozantinib group (21.1, not evaluable; HR 0.94; 95% CI 0.70–1.27; *p* = 0.69). Serious AE occurred more often with the triplet (126 of 262 patients, 48%) than with cabozantinib alone (84 of 256, 33 %) and events leading to death were reported in 17 patients (6 %) on the combination versus 9 patients (4 %) on monotherapy.

## 4. ICI-Related Immune-Related Adverse Events

ICIs are designed to activate the immune system against tumor cells; however, their use is frequently associated with irAEs, most commonly involving the skin, endocrine glands, gastrointestinal tract, lungs, and liver [92]. A retrospective, multi-center study with a minimum two-year follow-up in patients with mRCC treated with nivolumab monotherapy reported rash or pruritus as the most prevalent AE (22%, n = 16) of any grade [93]. Among grade ≥ 3 events, elevated hepatic enzymes were most common (5%, n = 4), followed by colitis or diarrhea (4%, n = 3). Overall, irAEs of any grade occurred in 45% of patients (n = 33), while 20% (n = 15) experienced grade ≥ 3 irAEs [93]. In turn, a systematic review and meta-analysis including 27 studies and 6,148 patients with RCC or urothelial carcinoma (UC) showed pooled incidences of irAEs at 44.2% (any grade) and 15.7% (grade ≥ 3) [94]. The antitumor efficacy of nivolumab is mediated through PD-1 pathway inhibition, which enhances T-cell activation and tumor recognition [95]. However, this immune stimulation may also lead to the recognition of self-antigens or the exacerbation of subclinical inflammation, resulting in irAEs such as endocrine dysfunctions, hepatotoxicity, colitis, and elevations in pancreatic enzymes [96,97,98,99]. Though uncommon, nivolumab has also been associated with serious irAEs, including pneumonitis, nephritis, myocarditis, and various neurotoxicities, the mechanisms of which are not yet fully understood [100,101,102,103]. Gastrointestinal toxicity generally results from immune-mediated inflammation of the bowel, manifesting clinically as diarrhea, nausea, or vomiting [97,98]. Pancreatic involvement may present as elevated serum amylase and lipase or pancreatitis, likely due to T-cell–driven inflammation of the pancreas [97,104].

Dermatologic irAEs are frequent and may result from immune recognition of skin antigens, leading to conditions such as erythematous macules, papules, plaques, pruritus, and vitiligo, typically affecting the trunk and extremities [105,106,107]. Renal adverse events, such as nephritis and proteinuria, may result from T-cell-mediated hypersensitivity or hapten-driven tubular injury, though precise mechanisms remain unknown [98,108,109]. Proteinuria can progress to nephrotic syndrome, characterized by peripheral edema, foamy urine, fatigue, and poor appetite [110]. Nephritis often presents with hematuria, decreased urine output, edema, and appetite loss, and both conditions may be accompanied by increased serum creatinine [95].

Neurological complications, though rare, may include posterior reversible encephalopathy syndrome and encephalitis. These conditions can present with similar clinical features, including headache, seizures, and altered mental status [98,111,112,113].

Cardiovascular irAEs are increasingly recognized, with myocarditis being the most frequently reported cardiac toxicity associated with ICIs [114]. Myocarditis may be associated with arrhythmias, myositis, or myasthenia gravis. Other cardiovascular toxicities include pericarditis, often with preserved left ventricular ejection fraction, and pericardial effusion without hemodynamic compromise [115,116,117,118]. ICIs have also been linked to various arrhythmias, including atrial fibrillation and sinus tachycardia as well as bradyarrhythmias such as atrioventricular block [118,119,120]. The additional cardiovascular complications reported include acute coronary syndromes, acute heart failure, Takotsubo syndrome, and cardiomyopathies [114].

Most irAEs resolve with ICI discontinuation and administration of corticosteroids or other immunosuppressants [92,121]. However, endocrine-related irAEs can result in irreversible damage, necessitating lifelong hormone replacement therapy. Examples include hypothyroidism following thyroiditis and adrenal insufficiency secondary to hypophysitis [92].

Interestingly, the occurrence of irAEs has been associated with improved clinical outcomes. A meta-analysis involving 27 studies found that patients who developed irAEs had significantly better PFS (HR = 0.44, 95% CI: 0.35–0.56, *p* < 0.01), better OS (HR = 0.47, 95% CI: 0.42–0.51, *p* < 0.01), an improved ORR (odds ratio [OR] = 3.59, 95% CI: 3.01–4.29, *p* < 0.01), and an improved disease control rate (OR = 4.23, 95% CI: 3.06–5.84, *p* < 0.01) [94]. These benefits were consistent across different tumor types and ICI regimens. Subgroup analysis suggested that mild to moderate irAEs, particularly those dermatologic or thyroid-related, were linked to superior survival outcomes. In contrast, pulmonary irAEs and severe (grade ≥ 3) events were associated with worse OS [94]. Several biological mechanisms may explain the association between irAEs and improved survival in patients treated with nivolumab and ipilimumab. One proposed mechanism involves molecular mimicry, wherein tumor and normal tissue share antigenic epitopes, triggering concurrent immune responses against both tumor and healthy tissues [122]. This process may potentiate antitumor immunity, especially in patients experiencing mild to moderate irAEs [123,124,125,126]. Elevated levels of pro-inflammatory cytokines such as interleukin-6 and interferon-gamma, which are key regulators of T-cell activity and immune cell recruitment, further link irAEs to enhanced antitumor effects. However, these cytokines also contribute to systemic inflammation and the development of irAEs [123,127]. Self-reactive T cells may infiltrate normal tissues, such as the myocardium, in cases of ICI-related myocarditis, reflecting antigenic overlap. B-cell activation and the generation of autoantibodies have also been implicated in irAE pathogenesis, with autoantibodies commonly detected in affected patients [123,127,128].

## 5. Current Role of ICIs in Clinical Practice

The management of advanced RCC has evolved significantly, with guidelines from organizations such as the National Comprehensive Cancer Network (NCCN), European Society for Medical Oncology (ESMO), and American Society of Clinical Oncology (ASCO) emphasizing the importance of risk stratification and appropriate treatment selection in both first-line and second-line settings [129,130,131].

Risk stratification is crucial in guiding treatment decisions for advanced RCC. The IMDC model is commonly employed to categorize patients into favorable-, intermediate-, or poor-risk groups based on clinical and laboratory parameters. This stratification aids in prognostication and informs therapeutic strategies [129,130,131].

The treatment recommendations for advanced or metastatic ccRCC vary based on patient risk profiles and previous treatments. For favorable-risk patients, first-line treatments typically involve combinations of ICI with VEGFR TKIs, such as axitinib + pembrolizumab, cabozantinib + nivolumab, and lenvatinib + pembrolizumab, with ipilimumab + nivolumab also recommended for those naïve to immune-oncology therapy. Second-line treatments are not specified for this group, suggesting the first-line therapies are generally effective.

For poor/intermediate-risk patients, similar first-line regimens are recommended, with an additional option of axitinib + toripalimab for intermediate-risk cases. Second-line treatments are not clearly defined for these patients either. In cases of progression after VEGFR TKI monotherapy, options like nivolumab or cabozantinib are suggested for second-line treatment. Additionally, combination treatments with two ICIs or ICI + VEGFR TKI are recommended for intermediate- or poor-risk patients as first-line therapy.

Overall, these guidelines emphasize the use of personalized, combination therapies in treating advanced ccRCC, with a focus on balancing the efficacy of immunotherapy and VEGFR TKI combinations [129,130,131]. The summary of guidelines is presented in Table 1.

## 6. Conclusions

First-line treatment of advanced ccRCC has been revolutionized by trials showing that combining immune checkpoint inhibitors with targeted agents clearly outperforms sunitinib monotherapy in efficacy, safety, and patient-reported outcomes. Combination therapies demonstrate varying degrees of success, which highlights the importance of deeper biological insights and more precise patient selection. Dual-checkpoint blockade with nivolumab and ipilimumab (CheckMate 214) utilizes two distinct mechanisms of T-cell activation—reviving exhausted effector cells in the tumor microenvironment via PD-1 inhibition and expanding the pool of tumor-reactive lymphocytes through CTLA-4 blockade, which together yield deep, durable responses and a clear survival plateau in intermediate- and poor-risk patients. However, this profound immune activation also precipitates a higher rate of immune-related toxicity and treatment discontinuation, suggesting that patients with less immunogenic tumors or lower risk may derive limited benefit relative to the added risk. By contrast, checkpoint-TKI combinations such as pembrolizumab plus axitinib (KEYNOTE-426) or nivolumab plus cabozantinib (CheckMate 9ER) benefit from synergy between VEGFR-driven vascular normalization, which improves T-cell infiltration and reduces suppressive myeloid populations, and the PD-1 blockade, achieving high response rates and broad efficacy across IMDC risk groups with more manageable chronic toxicities. However, even here, the outcomes are not the same. Tumors lacking pre-existing T-cell infiltrates, harboring mutations that reduce interferon signaling, or driven by alternative pro-angiogenic pathways (FGF, MET, AXL) often resist these strategies, while chronic VEGF inhibition can select for immunosuppressive myeloid phenotypes and collateral organ toxicity.

Trials of novel combinations, such as atezolizumab plus bevacizumab (IMmotion151), avelumab plus axitinib (JAVELIN Renal 101), or the lenvatinib-based regimens tested in CLEAR, present the same dynamic: dual targeting of immunity and angiogenesis can improve progression-free and OS, particularly in molecularly defined subgroups (for example, tumors with high T-effector and low myeloid signatures), but also carry burdens of overlapping toxicity that may offset quality-of-life gains, especially when OS benefits remain immature or marginal. 

The triplet approach of cabozantinib with nivolumab and ipilimumab (COSMIC-313) further enhances efficacy at the cost of controlling serious adverse events, while negative studies such as CONTACT-03 show that adding the PD-L1 blockade to TKI therapy in checkpoint-refractory disease can simply cause harm. Taken together, these diverse outcomes reflect the heterogeneity of tumor biology—variations in the immune microenvironment, angiogenic dependencies, and adaptive resistance mechanisms such as upregulation of alternative checkpoints (e.g., TIM-3, LAG-3), metabolic reprogramming (e.g., adenosine, IDO pathways), and engagement of redundant pro-angiogenic axes (FGF, MET, AXL). Traditional biomarkers like PD-L1 immunohistochemistry alone are insufficient to predict which patients will derive maximal benefit. Future progress will require multilayered biomarker strategies, integrating tumor gene-expression signatures (including T-effector, angiogenesis, and myeloid inflammation profiles), circulating tumor DNA to monitor emerging resistance mutations in real time, host germline factors such as HLA and Fcγ receptor polymorphisms, and potential microbiome compositions. Incorporation of functional imaging, such as PET tracers for PD-L1 or CD8+ T cells, and early pharmacodynamic readouts from peripheral blood or repeat biopsies will enable the timely adaptation of therapy. The utilization of these multidimensional biomarkers in clinical trial design and routine practice promises to match individual tumor biology to the combination most likely to achieve durable disease control with minimized toxicity, thereby ushering in a truly personalized era of combination therapy for advanced renal cell carcinoma.

## 7. Future Perspectives

As the therapeutic options expand, several avenues offer promise for improving outcomes in ccRCC. Improving outcomes for patients with ccRCC will depend on several key strategies. Advances in biomarker discovery and molecular profiling hold the potential to guide personalized treatment selection, enabling more targeted and effective therapies. Novel therapeutic combinations, including next-generation immune modulators and agents targeting hypoxia-inducible pathways, are under investigation and may offer enhanced clinical benefit. Optimizing the sequencing and duration of therapies remains a crucial challenge, particularly in the context of resistance to ICIs. The results of studies like CONTACT-03 highlight the limited efficacy of re-challenging with ICIs in certain settings, emphasizing the need for individualized treatment planning. As patient survival improves, quality of life is increasingly recognized as a central component of care. Integrating patient-reported outcomes into clinical practice will be essential for delivering truly patient-centered treatment.

## Figures and Tables

**Table 1:** ICIs in the ccRCC guideline comparison (NCCN, ESMO, ASCO).

Guideline	Risk Stratification (IMDC)	First-Line Treatment Recommendations	Second-Line Recommendations
**NCCN [129]**	**Preferred Regimens**	Favorable * Axitinib + pembrolizumab (category 1) Cabozantinib + nivolumab (category 1) Lenvatinib + pembrolizumab (category 1) Ipilimumab + nivolumab	**IO Therapy Naïve** None
		Poor/Intermediate * Axitinib + pembrolizumab (category 1) Cabozantinib + nivolumab (category 1) Ipilimumab + nivolumab (category 1) Lenvatinib + pembrolizumab (category 1)	**Prior IO Therapy** None
	**Other Recommended Regimens**	Favorable * Axitinib + avelumab	**IO Therapy Naïve** Axitinib + pembrolizumab Cabozantinib + nivolumab Ipilimumab + nivolumab Lenvatinib + pembrolizumab Nivolumab
		Poor/Intermediate * Axitinib + avelumab	**Prior IO Therapy** None
**ESMO [130]**	Local and locoregional ccRCC	**Intermediate–high- or high-risk operable ccRCC ** Adjuvant pembrolizumab [I, A; ESMO-MCBS v1.1 score: A].	
	Advanced or metastatic ccRCC	Favorable * Lenvatinib–pembrolizumab [I, A; MCBS 4] Axitinib–pembrolizumab [I, A; MCBS 4] Cabozantinib–nivolumab [I, A; MCBS 1] Ipilimumab–nivolumab [I, C; MCBS 4]	Further ICI therapy after first-line PD-1-targeted combination therapy is not recommended
		Intermediate- and poor * Lenvatinib–pembrolizumab [I, A; MCBS 4] Axitinib–pembrolizumab [I, A; MCBS 4] Cabozantinib–nivolumab [I, A; MCBS 1] Ipilimumab–nivolumab [I, A; MCBS 4] Axitinib–toripalimab [I,C]	
**ASCO** [131]	metastatic ccRCC	**Intermediate- or poor-risk disease** Combination treatment with two ICI, e.g., ipilimumab and nivolumab) or an ICI in combination with a VEGFR TKI; [Type: Evidence based, benefits outweigh harms; Evidence quality: High; Strength of recommendation: Strong].	Nivolumab or cabozantinib should be offered to patients who progressed on a VEGFR TKI alone [Type: Evidence based, benefits outweigh harms; Evidence quality: High; Strength of recommendation: Strong].
		Favorable risk * ICI in combination with a VEGFR TKI [Type: Evidence based, benefits outweigh harms; Evidence quality: High; Strength of recommendation: Strong]	
		**Select patients ** ICI monotherapy, e.g., pembrolizumab [Type: Evidence based, benefits outweigh harms; Evidence quality: Moderate; Strength of recommendation: Strong]	

* IMDC risk model; Abbreviations: ICI, immune checkpoint inhibitor; IO, immuno-oncology; TKI, tyrosine kinase inhibitor; VEGFR, vascular endothelial growth factor receptor.
